# On Vaccine Inoculation

**Published:** 1821-04

**Authors:** 


### Original Communications, Select Observations, etc.

### *On Vaccine Inoculation.* By Dr. KINGLAKE.

IT is much to be deplored that objections are cursorily taken up, and urged, against the anti-variolous power of vaccine disease, in a way that is far too capricious and illogical to be in any degree conclusive on the question. Every instance of apparent failure of vaccine inoculation in preventing small-pox, is regarded as bearing additional testimony against the influence of cow-pox in subduing the natural liability to the variolous disease. Instead of investigating the cases which are said to have failed with an earnest and diligent endeavour to detect the real state of the fact, too much credit is given to rumours on the subject, which, on close inquiry, are almost invariably found to be either wholly groundless or to rest on gratuitous assumptions.

The prevailing reputation of the insufficiency of vaccine inoculation for the purpose for which it is instituted, is in this neighbourhood such as almost to challenge the possibility of defending its efficacy from the wide-spreading opposition with which it is assailed. It is obvious that those who are chiefly occupied in opposing the vaccine practice, are persons who are not competent to examine the subject on any scheme that could confer validity on the unfavourable opinion that is adopted respecting it. Popular feeling on a medical subject, is more likely to be erroneous than correct; and, whatever may be the current of general belief on these occasions, it can scarcely be restrained by reasoning or counteracted by facts.

The vaccine practice had to contend originally against prejudices of long and established growth. The general feeling was in favour of inoculated small-pox, which, compared with the natural disease, was a greatly diminished and a very manageable affection. It was hardly probable that this great practicable benefit would be relinquished, to substitute in its stead a novel and an untried mode of effecting a similar object of security by means apparently far-fetched and arbitrary, or, at least, not intelligible to the public understanding. In this state of change and abandonment of an old acquaintance, it was not extraordinary that disappointment should be anticipated, and that occurrences at all savouring of uncertainty or indecision should be precipitately regarded as proofs of failure and insufficiency. Thus is the popular feeling at the present moment disposed to condemn and reject the vaccine practice, and to accumulate, in the most vague and questionable manner, testimonies against its anti-variolous influence.

The rumours of insufficiency in the vaccine disease as a permanent security against the infection of small-pox, are extensive, and propagated with a degree of confidence that will require the utmost address of the medical faculty to repress and overcome. These reports have certainly originated in misapprehension of the nature of the disease that has imposed itself for small-pox. It has been erroneously imagined that the vaccine disease was to afford an exemption from almost every variety of herpetic complaint: whenever, therefore, an eruptive distemper has occurred, it has been, by the aid of the most equivocal and fanciful resemblances, misconstrued into small-pox. Essential differences as to the appearance, stages, and duration of the disease, have been excluded from consideration, for the purpose of deciding on the identity of variolous affection. In the persuasion that every cutaneous eruption, whether confluent or distinct, must be small-pox, scarcely a disease of the skin can occur, whether affecting the general health or not, or however dissimilar its progress may be to that of small-pox, but it is indiscriminately held to be variolous; for no better reason than that the patient had been vaccinated, and yet the skin retains a susceptibility for eruptive malady!

The delusion into which the public mind has been betrayed by the occurrence of eruptive maladies in vaccinated persons, affords no sort of warranty for the opinion beginning to prevail, that the alleged vaccine preventive is fallacious and unworthy of confidence. The instances of the reputed failure of the anti-variolous power of vaccination, must be cited with the utmost minuteness; all the attending circumstances should be stated; and an analogy be made out, of the most strict and undeniable description, before either the want of vaccine efficacy, or the identity of variolous disease, can be justly recognized and admitted.

It has occurred to me to see many of the cases affirmed to be variolous after vaccination, but in no one instance has the affection appeared to be sufficiently genuine to authorize a conclusion that it was really small-pox. The resemblance was but very faint in any particular circumstance; if, indeed, it could be considered as presenting an outline of the disease, the filling-up, so as to afford it a cognizable likeness, would be a work of imagination, wholly inconsistent with the natural appearances sought to be imitated.* Things which are naturally different cannot be identified; the subsisting dissimilarities will be shown in corresponding aspects, which never can assume a similitude amounting to sameness.

It is probable that the vaccine disease, supplied as it is by a morbid process taking place in the animal economy of the cow, is in reality of the same kind as the variolous affection occurring in the human species of animal life, and that the two vary only in the temperaments of the different animal organizations in which they are respectively generated. The variolous disease may as well originate in the cow, or any other animal, as in the human frame, whether its source be in any relative conditions of the atmosphere, or in any latent properties connected with the standard health of the vaccine animal. The disease may also be extended to the cow by contagion. The warm head of a person infected with the small-pox might, in the act of milking, impart the disease to the cow; or it might, in turn, be communicated to the milker of that animal. In either case a disease results, which in the cow is called cow-pox, and in the human species small-pox. A difference fully equal to what is present may be supposed to arise in the same disease, occurring in animals so dissimilarly constituted, nourished, and habituated, as the human and brute kinds of organic life are. If the diseases be essentially alike, differing only in accidental circumstances, then it is natural that, if the one could be trans-

* *December the 10th.*—A case of varioloid disease after vaccination has been just shown to me in this town, that approaches nearer to the true variolous character in its confluent form than any I had previously seen. The eruptions on the face had every where ran into each other, and had induced as large and as unsightly an intumiscence of the whole countenance, as occasionally happens in the most violent degree of natural small-pox. The pustules on other parts of the body were distinct, less than the true variolous size, and without any central depression. The contents of the eruptions were puriform, and even the parts in both arms where vaccine inoculation had been some years before performed, were clustered with pustules. The throat also had been sore, and loaded with an inordinate secretion of mucus. But, with this aspect of real variolous disease, the constitutional excitement was not seriously disturbed. The febrile action was restrained, and the whole of the morbid process exhibited a mitigated and altered character from that which would have attended unvaccinated small-pox. Violent as this case appeared to be, it still seemed to be divested of the genuine variolous virulence, which probably had been so subdued or modified by vaccine influence as to have rendered a disease comparatively devoid of danger, which, in the natural and unchanged state, would have been imminently hazardous, if not almost inevitably destructive of life.

ferred from the brute to the human frame, it would neutralize or destroy the inherent susceptibility for subsequently taking the kindred affection, and thus prove an effectual preventive, such as the vaccine disease is held to be.

It probably would be impossible to induce in the animal excitability of the cow a disease closely resembling the variolous ailment, the nature of the animal being such as, perhaps, to be insusceptible of the aggravated form which that affection assumes in the human species; yet the attenuated and mild form under which the disease appears in the cow may be nevertheless the peculiar malady that constitutes small-pox, and, in as far as it is communicable, it avails in securing against either its spontaneous or artificial production in the virulent aspect under which it is apt to show itself in the human frame. If the diseases called vaccine and variolous possess radical identity, of which there appears to be much probability, the only question as to the preventive power that the one has over the other, of practical importance, is, whether the vaccine disease, or, in other words, the small-pox occurring in the cow, is not too mild, too destitute of virulence, permanently to destroy the susceptibility existing in the human frame for being affected by the variolous disease.

If the natural capability for being infected by small-pox can only be subdued or extinguished by a degree of morbid impression that could never wear out, then it may be reasonably supposed that an highly-attenuated or weakened state of the disease, whether resulting from the animal economy of the cow or any other animal, might furnish an insufficient security against future infection. The diseases would appear to be, in radical quality, one and the same; if, therefore, the milder or vaccine form be preferable to the human or variolous character, it deserves to be adopted under such precautions and observances as would be likely to render its desired influence infallible.

The utmost caution is necessary in the practice of vaccination, to ensure the preventive influence against small-pox that is intended to be produced. If the vaccine disease has only a power of destroying susceptibility for variolous affection when it is applied in its most genuine state, it behoves the advocates and promoters of the substitute to look narrowly to that real quality by which alone the object in view can be accomplished. It should be sedulously noticed if there be, in the affection that has been induced, any variations, discrepancies, or incongruities that did not accord with the customary and characteristic form of the vaccine disease. The matter that is furnished by a vaccine pustule might, when inserted under the skin, induce inflammatory excitement, and that too of the areolous character, that would have very much the aspect of the real disease; yet it may not come up to the standard for destroying the inherent susceptibility for small-pox; or, to be more explicit, it may not be the vaccine modification of small-pox that will prevent the human form of that malady from subsequently occurring.

Much assiduity and nice discrimination have been employed in delineating the precise state and character of the vaccine disease, that would counteract or resist a future appearance of variolous affection; and the directions given on this subject are sufficiently copious, practical, and efficient, to answer every useful purpose. The limpid state of the fluid resulting from vaccine inflammation, is that in which the most active and most anti-variolous quality of the disease is exerted. If the fluid be suffered to advance to the purulent state, the specific properties on which its genuine influence depends are either deteriorated or wholly annihilated. Infectious fluids acquire the power of communicating infection from a precise arrangement of their constituent parts. The vascular action of organic life, under circumstances issuing from the combining and arranging processes of animal chemistry, will furnish material power capable of producing a specific effect; and this power can only be known by the peculiar influence which it is enabled to exert.

Vaccine matter, from the time of its insertion under the skin until its final exsiccation, may be capable of producing a vast variety of disease, if employed at all the different stages of maturation; but it is well ascertained that it is only at a particular period of this process that the product has the power of exciting the peculiar disease that eradicates the native susceptibility for taking variolous affection. The numerous failures and anomalies that have occurred in the practice of vaccination, have chiefly resulted from the inoculation having been performed with matter destitute of the specific properties necessary for generating the real disease. Local inflammation, suppuration, and eschar, have been the demonstrable signs of final vaccination; but, unhappily, in many instances, they have not eventually proved the true *criteria* of efficient vaccination. The practical difficulty, and indeed objection, attending vaccine inoculation, is the precise and limited, though in different constitutions often variable, period, at which matter possessing the specific quality of the disease should be taken. The progress of inflammatory action at the place of insertion is not in all temperaments the same: in some it is earlier than in others, which requires that an experienced and discriminating observation of the actual state should at all times be made, to obviate the fallacies that would be likely to result from an imperfect condition of that fluid. To provide against the degeneracies to which all specific arrangements of animal substances are liable, when detached from the circumstances in which they originate, it would appear to be advisable not to rely for an indefinite period on the vaccine matter that had been transferred through a very extended succession of human subjects. The diversity of temperamental influence that may have been exerted on it, may possibly disturb and lessen the precise condition of activity and power on which its anti-variolous efficiency depends. Recurring, therefore, at short intervals to the cow, the genuine source of supply, for fresh and unaltered matter, would be the best security against the diminished power, and eventual insufficiency, of a fluid that may have become, in some measure, effete and inert, by having been too long employed in human inoculation.

The reports of failures of the protecting influence of cow-pox against small-pox, have been so extensive and so various as to have ideally furnished almost every conceivable diversity that could occur in the variolous disease ensuing the vaccine inefficacy. It has been affirmed, that unmitigated and unaltered small-pox has presented in all its terrors; that the worst forms, and most inveterate symptoms, of that disease have arisen after vaccination. Hear-say authority on medical, as well as on all other subjects, is questionable, and should never be cited in justification of what is merely asserted to be true. It has not occurred to me to see an instance in which vaccine influence had been exerted in the system, where the aspect of the variolous disease that afterwards shewed itself sufficiently resembled the genuine character of that affection, to admit of regarding it as that ailment. That an approach may be made to the appearance of a given disease, is very probable: indeed, the radical similarity of most diseases, and the apparent affinity of many of them, must necessarily furnish the sort of general similitude that might go to particular instances of sameness; but this is not the identity that is insisted on, as obtaining between original small-pox and that affection which occasionally happens after vaccination. Vaccinated small-pox, if the phrase may be allowed, is so modified, so reduced in virulence, and so circumstanced in appearance, as to be essentially different from the primary and unneutralized disease.

If small-pox, in its natural and unadulterated character, can either be prevented, or so changed in its nature as to be divested of its virulence and rendered comparatively mild and harmless, the great object aimed at in substituting for it vaccination is answered; and, although the cutaneous surface, and even an irritable temperament, may not be secure against subsequent variolous excitement, yet it will be unaccompanied with danger, and, of course, not deserving any serious apprehension. It could not be justly imagined that a disease like that of smallpox could ever be so annihilated in its power as to be incapable of exciting the irritable conditions of vital action; but, if that excitement be devoid of risk and attended with no considerable inconvenience, the affection has been disarmed of its malignity, and mankind have derived all the exemption from its destructive ravages that vaccination, or any other substitute, could have, reasonably, been expected to afford.

The anti-variolous power manifested by vaccine disease, has succeeded to an extent that evinces a certain force of that power, and proves that, were it applied in all the relative circumstances necessary to render it efficient, it would be as nearly infallible in its influence as the variable nature of diseased action will admit. If, in a majority of instances nearly reaching the whole, the vaccine disease be found to prevent the occurrence of small-pox, can it be justly held that it is an insufficient and an insecure substitute for that affection? It may be undeniably affirmed, that the examples of entire failure of the vaccine over the variolous disease are so few and so equivocal, as to afford no valid objection to the practice of vaccination. The benefit derived from substituting cow-pox for small-pox, is that of having a mild disease instead of one that is in its nature virulent, and occasionally attended with extreme danger. If, then, this amelioration can be obtained in a morbid excitement to which there is a natural liability, is it not a positive advantage, a comparative good, worthy of preference, and deserving of fearless adoption?

Vaccine disease may not exempt the skin from eruptive affections, nor does it seem capable of altogether extirpating the inherent susceptibility for variolous excitement, but it fails not to lay the disease under such restraints, and so to modify its power, as almost to annihilate its force and character. When it supervenes vaccine disease, its onset is slight, its duration short, and the attending pustular inflammation is in general insufficient for the suppurative process. The eruptions appear in diminished size, remain visible only for a few days, and then finally vanish. There is but little or no concomitant fever; indeed, hardly sufficient, generally speaking, to disturb either the healthy sensation or function of any vital organ. The alarm, then, would appear to be groundless concerning the incompetency of vaccine to subdue the natural susceptibility for variolous disease. It is fully equal to that effect with regard to all that can be correctly considered as specific, peculiar, and exclusively constituting the original nature and form of that affection. Persons who have undergone the morbific process of vaccination are, with respect to the variolous disease, in a state of security very similar to that of those who have been subjected to inoculation for small-pox. The efficiency of variolous inoculation, like that of vaccination, will much depend on the specific properties of the matter employed for communicating the disease; and, when the affection has fully taken place, a new infection, to a limited extent, may possibly occur. The skin will take on the variolous excitement in two or more instances; but then, as after vaccination, it is in so subdued a form as not to exhibit the genuine character of original disease. Much obscurity exists in the laws by which inherent susceptibility for being only once affected by certain specific diseases, is governed. It seems to be the property of all substances exerting a stimulant agency in the animal economy, to become diminished in power by repetition and long continuance. Diseased excitement of every variety evinces this property to a given extent, but in particular instances, as small-pox, cow-pox, measles, whooping-cough, &c. it is more strikingly and positively exemplified. The primordial aptitude, therefore, for taking on disease, as shown in the innate susceptibility for small-pox, would appear, if not wholly extinguished by either variolous or vaccine affection having once actually occurred, to be at least so reduced and altered as not to admit of a state of disease that could assume any serious degree of constitutional violence.

Enough has been noticed of the nature and character of vaccine disease to remove all doubt as to its efficacy in preventing small-pox. Indeed, the proofs of this power have been so abundantly conclusive, that it would be superfluous to insist on a fact so obvious and undeniable. That which has occurred, therefore, in a manner so indubitable, will take place again in similar circumstances; so that natural correctness will be found to be perfectly steady and undeviating, whatever may be the vacillation and inconsistency of speculative opinions. Were the laws of nature to yield to the unreflecting impatience, to the prejudiced notions, and to the erroneous calculations of man, instability and disorder would soon predominate over fixation and harmony. Knowledge can only be acquired by patient and accurate observation. When the closest attention to facts has ascertained unquestionable truths, neither surmises nor rumours, however speciously authenticated, can invalidate the verity of such intelligence. The inadequacy of vaccine disease to supplant the inherent liability to small-pox, may be affirmed and maintained under a large share of apparent proof; but, if the natural law that governs the question at issue should oppose such a conclusion, it will be unavailing to attempt to withstand its irresistable validity.

The varioloid affection that has occurred, in numerous instances, after vaccination, as well as after small-pox itself, differs widely in appearance, and probably also in reality, from genuine variolous disease. The opinion that varicella, or chicken-pox, has the same origin as small-pox, or that they are only different modes of the same malady, is not warranted by the one form of ailment preventing that of the other. The most violent degree of small-pox does not incapacitate the system for being affected by chicken-pox; nor does the latter destroy the natural susceptibility for assuming the former. They cannot interchange and become a substitute for each other, as might be effected if they were one and the same disease, and drew their respective existence from the same source. Unlike the vaccine fluid, the vesicular lymph of the chicken-pox will not, by inoculation, at all affect the natural liability for taking small-pox. Were the diseases identical, that would be the case; for, whether the one or the other form be brought into effect, the ground or nature of the affection would be similar. The influence of vaccination in obviating a subsequent accession of variolous disease, justifies the inference of there being at least an analogy subsisting between the diseases, approaching so nearly to identity, if not positively the same, as to render an attempt to distinguish them extremely difficult, and, in a practical consideration, unimportant.

If there be any varioloid quality in chicken-pox, it is too ineffective to produce any of the characteristic appearances and influence of the variolous disease. The intensity and quality of excitement necessary to generating the specific powers of small-pox have not existed in chicken-pox; it is, therefore, a state of disease varying too widely from variolous affection to admit of being correctly denominated a milder form of the same ailment. The vaccine malady has strikingly furnished a substitute for variolous disease, that either wholly supersedes the natural disposition to that complaint, or so lessons its original violence, as to change it into a disorder of no comparative danger. The pustular and vesicular characters of variolous and varioloid affections, whether ensuing vaccination, chicken-pox, or small-pox itself, evince the different degrees of inflammatory excitement attending those dissimilar forms of disease. The pustular inflammation of the small-pox is of the phlegmonous kind, and is sufficiently powerful to induce suppuration, and to furnish the purulent fluid contained in the pustules: in the vacciolous and varicellous vesicles, the concomitant inflammatory action is of an erysipelatous nature, and terminates in a limpid effusion under the cuticle, and not in maturation. These differences are strongly marked, and demonstrate a variation in the force and condition of the morbid action obtaining in the several cases, that proves a very dissimilar state of disease. Were there a radical identity subsisting between the three different forms of disease distinguished by the several names of variola, vacciola, and varicella, and the mildest of them could be substituted for the most violent, it would be (as appears in the adopted practice of vaccination) an enviable advantage to prefer, in a choice of evils, that which would be least injurious in exerting a preventive influence against a severer form of disease.

If varicellous affection were as unequivocally influential in subduing the innate susceptibility for small-pox as vaccine disease, whether happening naturally or induced by inoculation, it would be just to regard it as another modification of variolous malady; and it would be immaterial which of the two should be adopted as a substitute for the more violent disease sought to be prevented. Whether chicken-pox be in its nature an original and distinct disease, or whether it be an offspring of small-pox, is but of small moment, compared with the decisive fact of its not being capable of counteracting the usual occurrence of that affection. If it really possesses an affinity to small-pox, it is too remote and inoperative to admit of being an effectual obstacle to that disease, and must therefore, with reference to any protecting power that it may have against small-pox, be considered as an independent distemper. Vaccine disease is characterized by the strongest kindred resemblances to small-pox, and has, in a degree equally extensive and gratifying, proved either an adequate bar against that affection, or has so altered and modified the natural susceptibility to it, as to have rendered its eventual occurrence exceedingly moderate, safe, and manageable.

Considering vaccine inoculation both retrospectively and prospectively, looking to its merits and demerits from all that has yet occurred on the subject, its just claims would appear to be imperative on the intelligent and humane advocates of that salutary practice, not to suffer their well-founded confidence to be in any degree shaken by the prevailing clamours and prophecies concerning its inefficacy in lastingly preventing the accession of small-pox. If popular doubts and alarms should be in any measure countenanced, or even connived at, by medical practitioners, the result will soon be irreparably injurious to the just pretensions of vaccine inoculation. Happily for the vital interests of mankind, the real anti-variolous power of the vaccine disease has not yet been impugned in a way to prove that efficient vaccination is not almost invariably preventive of small-pox; and that, when it does not altogether counteract the occurrence of that disease, it so changes its character, and so divests it of virulence, as to cause it to appear under a form too mild and tractable,—in fact, too slightly varioloid,—to warrant any anxiety for its termination.

The contributions made to the general amount of mortality by the destructive ravages of small-pox previously to the introduction of the vaccine preventive, are truly appalling, and should elevate the best feelings of philosophy in defence of a practice that either wholly supersedes the small-pox, or modifies that mortiferous disease into a comparatively safe and harmless malady.

Medical practitioners have vast power in controlling and directing public feeling on the momentous subject of vaccination. All difficulties, all anomalies, occurring in the practice, should be, if possible, satisfactorily explained. No questions of uncertainty and solicitude should remain unanswered. Ample experience has been already afforded of the preventive power of the vaccine over the variolous disease, and its title to unsuspecting confidence would appear to be too well established to justify any reasonable distrust in its capability of either wholly resisting the occurrence of small-pox, or of so mitigating its virulence as to render it an affection of scarcely any calculable importance.

*Taunton; December* 15*th,* 1820.

